# AmrZ is a global transcriptional regulator implicated in iron uptake and environmental adaption in *P. fluorescens* F113

**DOI:** 10.1186/1471-2164-15-237

**Published:** 2014-03-26

**Authors:** Francisco Martínez-Granero, Miguel Redondo-Nieto, Pilar Vesga, Marta Martín, Rafael Rivilla

**Affiliations:** 1 Departamento de Biología, Universidad Autónoma de Madrid, C/Darwin, 2. 28049 Madrid Spain

**Keywords:** AmrZ, Transcriptional regulator, Iron, *Pseudomonas*, ChIP-seq

## Abstract

**Background:**

AmrZ, a RHH transcriptional regulator, regulates motility and alginate production in pseudomonads. Expression of *amrZ* depends on the environmental stress sigma factor AlgU. *amrZ* and *algU* mutants have been shown to be impaired in environmental fitness in different pseudomonads with different lifestyles. Considering the importance of AmrZ for the ecological fitness of pseudomonads and taking advantage of the full sequencing and annotation of the *Pseudomonas fluorescens* F113 genome, we have carried out a ChIP-seq analysis from a pool of eight independent ChIP assays in order to determine the AmrZ binding sites and its implication in the regulation of genes involved in environmental adaption.

**Results:**

154 enriched regions (AmrZ binding sites) were detected in this analysis, being 76% of them located in putative promoter regions. 18 of these peaks were validated in an independent ChIP assay by qPCR. The 154 peaks were assigned to genes involved in several functional classes such as motility and chemotaxis, iron homeostasis, and signal transduction and transcriptional regulators, including genes encoding proteins implicated in the turn-over of c-diGMP. A putative AmrZ binding site was also observed by aligning the 154 regions with the MEME software. This motif was present in 75% of the peaks and was similar to that described in the *amrZ* and *algD* promoters in *P. aeruginosa*. We have analyzed the role of AmrZ in the regulation of iron uptake genes, to find that AmrZ represses their expression under iron limiting conditions.

**Conclusions:**

The results presented here show that AmrZ is an important global transcriptional regulator involved in environmental sensing and adaption. It is also a new partner in the complex iron homeostasis regulation.

## Background

AmrZ is a regulatory protein belonging to the Arc family of regulators that contains a Ribbon-Helix-Helix (RHH) DNA binding domain. It is encoded in the genomes of all strains of the genus *Pseudomonas* sequenced so far. AmrZ appears to be an important regulator in *Pseudomonas aeruginosa*, since a null mutant is severely impaired in a murine *in vivo* pathosystem, being displaced by the wild-type strain [1]. AmrZ was originally described as AlgZ, because of its role in the regulation of alginate synthesis in *P. aeruginosa*[2]. In this bacterium, AmrZ is essential for alginate production and is required for transcriptional activation of *algD* expression [3]. Therefore, conversely to other RHH proteins that are transcriptional repressors, AmrZ was originally identified as an *algD* activator. However, it has been shown that AmrZ binds to its own promoter repressing its transcription [4]. It has also been shown that AmrZ represses the production of the aggregative polyssacharide Psl, which is implicated in biofilm architecture [5,6]. The repression of Psl production occurs at the transcriptional level, by binding to a region overlapping the *pslA* promoter [7]. Therefore AmrZ is an atypical RHH protein possessing both transcriptional activation and repression activity. The X-ray crystal structure of AmrZ binding to the *amrZ* and *algD* promoters has shown that AmrZ uses different interactions for repression and activation [8].

Expression of *amrZ* depends on the sigma factor AlgU (previously known as AlgT) [2,9]. AlgU itself is implicated not only in alginate production, but it is required for the environmental control of the expression of multiple virulence and adaption traits in several species of pseudomonads [10-13] and it is considered a general stress response sigma factor [14,15].

Besides exopolysaccharide production, AmrZ is implicated in the regulation of motility. AmrZ controls twitching motility (a pili dependent motility) and pili biogenesis in *P. aeruginosa* in a way that requires the DNA binding activity [16]. However, in this case, the precise function of AmrZ as a transcriptional regulator has not been determined. Flagella mediated motility is also regulated by AmrZ. In mucoid *P. aeruginosa* strains, such as those which appear in the lungs of cystic fibrosis patients, AmrZ represses the transcription of the master flagellar regulatory gene *fleQ*, resulting in aflagellated cells [17]. Regulation was shown to involve binding of AmrZ to the *fleQ* promoter, although conversely to the *algD* and *amrZ* promoters, the precise protein-DNA interactions have not been determined.

*Pseudomonas fluorescens* F113 is a biocontrol strain [18] and a model for plant rhizosphere colonization [19-21]. In this strain, AmrZ regulates swimming motility by downregulating the expression of *fleQ*. F113 *amrZ* and *algU* mutants show a hypermotile phenotype caused by overproduction of flagella components [22]. Hypermotility in this strain has been related with enhanced competitive colonization of the rhizosphere [23] where it has been shown to be more important than the ability to form biofilms [19]. Considering the importance of AmrZ for the ecological fitness of pseudomonads and taking advantage of the full sequencing and annotation of the F113 genome [24,25] we have performed a ChIP-seq analysis, in order to determine the AmrZ binding sites and its possible regulatory roles.

## Results and discussion

### AmrZ binds to multiple sites in the F113 chromosome

**Figure 1:**
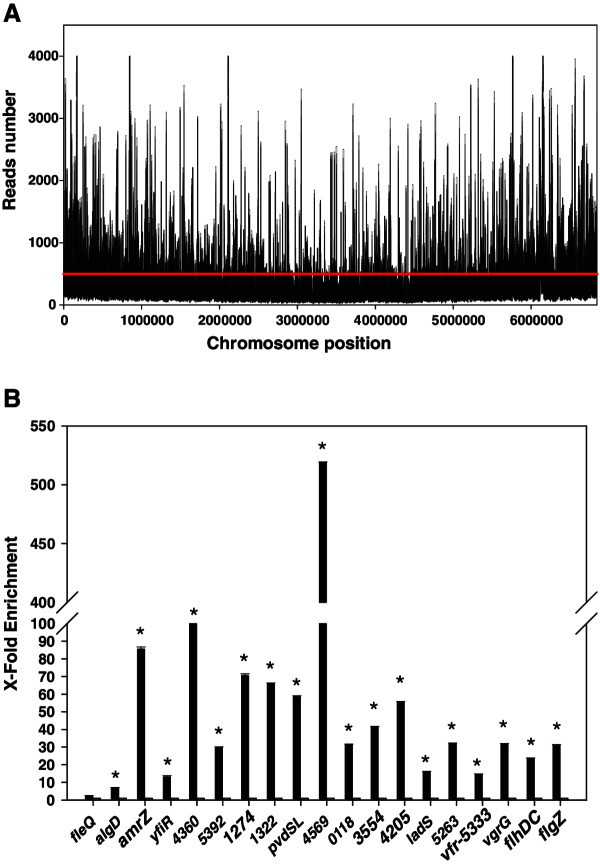
**ChIP-seq analysis of AmrZ binding sites in the *Pseudomonas fluorescens *F113 chromosome. A**. Distribution of putative binding sites along the F113 chromosome. Y axe represents the number of readings on each chromosome position. X axe represents the chromosome position with a window of 100 nucleotides. Red line marks the average number of readings plus standard deviation. **B**. ChIP analysis of selected enriched regions. 18 regions that appeared enriched in the ChIP-seq analysis were tested in an independent ChIP assay by qPCR. Selected regions corresponded to regions upstream of genes or ORFs as indicated in the x axe. Numbers correspond to PSF113 annotation. Y axe represents fold enrichment of immunoprecipitated DNA (black bars) against a mock control (gray bars). The region upstream the *fleQ* gene, that did not appeared as enriched in the ChIP-seq assay was also included in the analysis. The asterisks denote statistically significant differences with the mock controls (p < 0.05).

10 ng of immunoprecipitated DNA pooled from 8 independent ChIP assays (4 with N-tagged and 4 with C-tagged AmrZ-HA) were used to generate an Illumina library. Sequencing of the library yielded 12686888 sequences of 75 nucleotides with over 90% of sequences above Q30 quality. Around 93% of the original reads were conserved after trimming and cleaning (see Methods) and 94% of the former sequences aligned with the genome sequence of *P. fluorescens* F113. By defining a stringent cut-off (score ≥1000, fold enrichment ≥5.5), 154 peaks were detected, suggesting that AmrZ is a global regulatory protein. Figure 1A shows the distribution of these peaks along the F113 genome, showing that the peaks are not concentrated in specific regions but uniformly distributed along the whole chromosome. 117 of the peaks (76%) were mapped to intergenic regions. Since the percentage of intergenic regions in the F113 genome is of only 13%, the results show a very strong bias of the putative AmrZ binding sites to intergenic regions where most promoter regions lie, suggesting again the role of AmrZ as a transcriptional regulator. Because of gene orientation, for most intergenic regions (98.3%), the peak could be assigned to the upstream region of at least one gene. When only one gene was located in an appropriate orientation, the binding site was assigned to this gene. In the case of peaks located in intergenic regions between two divergent genes, the binding site was initially assigned to both genes. Additional file 1 shows the assignation of peaks to genes. It is interesting to note that peaks were detected upstream of its known targets in *P. aeruginosa*, *algD* and *amrZ*[2,3]. However, no peak was found upstream of *fleQ*, whose promoter region has been shown to bind to AmrZ in *P. aeruginosa*[17]. Since in *P. fluorescens* F113, AmrZ represses *fleQ* expression [22] the transcriptional regulation of *fleQ* by AmrZ should be indirect in this strain.

In order to validate the peaks, an independent ChIP assay was performed. Primers designed to amplify 18 of the detected peaks, including the peaks located upstream of *algD and amrZ*, were used in qPCR assays (Figure 1B). The region upstream of *fleQ* was also included in the assay. The *dif* region was used as a negative control. As shown in Figure 1B, this assay validated all the peaks detected by ChIP-seq and confirmed the lack of a peak upstream of the *fleQ* gene, indicating again that in F113 the transcriptional regulation of *fleQ* by F113 is indirect.

### AmrZ putatively binds to a conserved DNA motif

**Figure 2:**
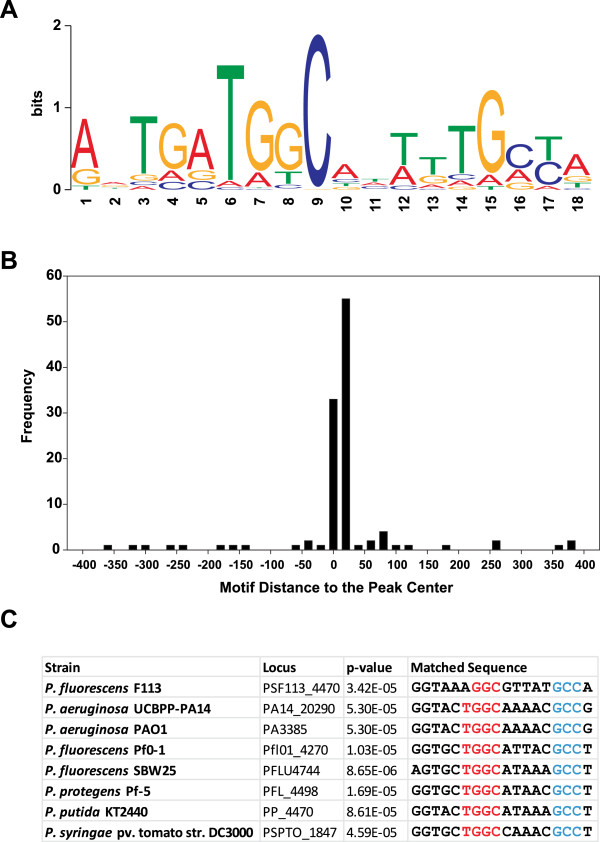
**Identification of the AmrZ binding site. A**. MEME logo identified as the AmrZ binding site. This motif is present in 76% of the enriched regions identified by ChIP-Seq. **B**. Frequency distribution of the distance of the motif to the peak center. The center of the peak was determined as the nucleotide(s) with the maximum reads within each region. A window of 20 nucleotides was used. More than 90% of the motifs were located within 50 nucleotides from the peak center. **C**. Alignment of the AmrZ binding site located upstream the *amrZ* gene in selected pseudomonads’ genomes. The two GC rich regions within the motif are indicated in red and blue. P value for all the motifs with respect to the MEME logo is indicated.

In order to determine the putative binding sites of AmrZ, the 154 regions that contained peaks above the defined threshold were analyzed with the motif finder pipeline MEME. As shown in Figure 2A, a putative motif was detected (E-value 1.7e^−53^). This motif was present in 115 (74.6%) of the peaks (Aditional file 1) and was generally located in a central position within the peak (Figure 2B). The motif is present upstream the *amrZ* and *algD* genes that have been shown to bind AmrZ in *P. aeruginosa* by gel retardation assays [3,4]. As shown in Figure 2C, this motif was congruent with the AmrZ binding site demonstrated by crystallographic analysis in the *amrZ* promoter in *P. aeruginosa*[8,26], showing a p-value of 5.30E-05. The motif is also highly conserved in other pseudomonads confirming that it corresponds to an AmrZ binding site.

### AmrZ regulates the expression of multiple genes in *P. fluorescens*

**Figure 3:**
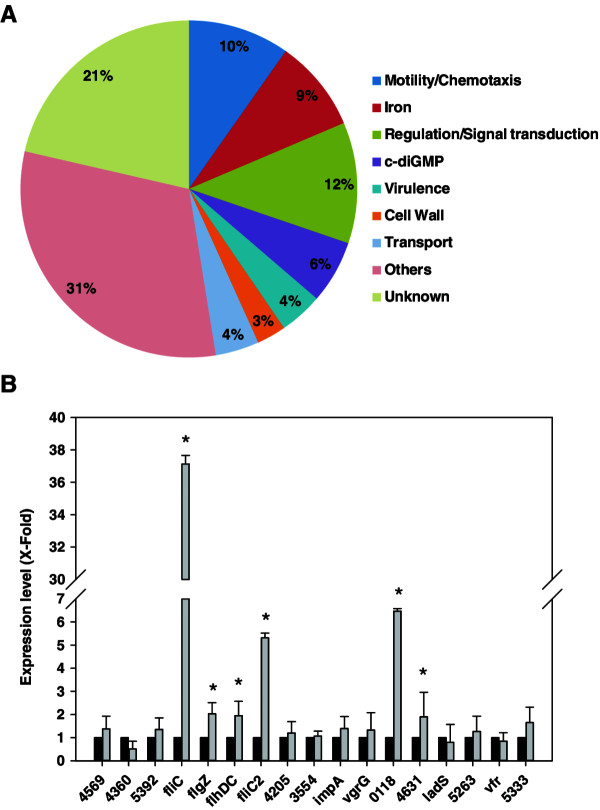
**AmrZ regulates a large number of genes, many of them implicated in environmental adaption. A**. Pie chart representation of genes potentially regulated by AmrZ, distributed by functional classes. Genes in this chart are described in Additional file 1. Functional classes were assigned by ontogeny analysis. **B**. Expression analysis of selected genes in *P. fluorescens* F113 and an *amrZ* mutant background. Relative expression (X-fold) in the *amrZ* mutant (gray bars) against F113 (black bars) is shown. Numbers correspond to PSF113 annotation. Expression was determined by qPCR. RNA was extracted from cells growing in LB medium at 0.8 O.D._600_. Asterisks denote statistically significant differences, p < 0.05.

Gene ontology and InterPro analysis of the 215 genes to which peaks had been assigned (Figure 3A and Additional file 1) showed that several functional classes such as motility and chemotaxis, iron homeostasis, signal transduction and transcriptional regulators were overrepresented.

AmrZ was previously shown to regulate motility in *P. aeruginosa*[17] and *P. fluorescens*[22], mainly through repression of the gene *fleQ*, which encodes the master regulator of flagella synthesis. In this analysis, 21 motility related genes have been assigned to peaks. Although *fleQ* has not appeared in the analysis and does not show the motif in its promoter region, genes related with the synthesis and function of the two flagellar systems present in *P. fluorescens* F113 such as *flgZ*, *flhDC, fliC2* and *flgM2* are among those putatively regulated directly by AmrZ. Besides these genes, the analysis has also identified as potential AmrZ targets genes related with chemotaxis, especially 13 genes encoding methyl accepting proteins (MCPs). This represents almost 50% of the MCP encoding genes present in the F113 genome [25].

Genes related to iron homeostasis were also overrepresented. 19 identified genes, included genes for the production of the siderophore pyoverdine (*pvdS*, *pvdL*), genes encoding iron uptake related extracytoplasmatic sigma factors (ECFs) and siderophore receptors: *fecI*, *tonB*, among others. These results suggest that besides regulating alginate production and chemotactic motility, AmrZ is a major regulator of iron homeostasis.

The analysis also showed 38 genes implicated in regulation and signal transduction as potential AmrZ targets. Interestingly, one of them is *algU* which encodes a sigma factor required for *amrZ* transcription [9] and an important node in the regulation of motility [22] and environmental adaption [27-29]. If under some circumstances, AmrZ transcriptionally regulates *algU*, this regulation would increase the complexity of AlgU regulation. It has been previously shown that *algU* is transcriptionally regulated by SadB and post-transcriptionally by the GacAS system [22]. AlgU is also regulated at a post-translational level by antisigma factors and directed proteolysis [30-32].

Within the signal transduction functional class, a clear overrepresentation was observed for genes encoding proteins implicated in the turn-over of the signaling molecule c-diGMP, including 13 genes encoding putative diguanylate cyclases and/or c-diGMP specific phosphodiesterases. c-diGMP is implicated in lifestyle transitions, such as sessility vs. motility or acute infection vs. chronic infection (reviewed in [33]) being frequently dependent on environmental sensing.

Taken together these results indicate that AmrZ is likely to be an important transcriptional regulator implicated in environmental sensing and adaption. The fact that an important number of regulatory and signal transduction proteins are among the potential targets for AmrZ regulation, indicates that the AmrZ regulon could be very large. The importance of AmrZ is also reflected in its conservation along the genus *Pseudomonas*.

In order to analyze the role of AmrZ in regulation, 16 of the genes identified as potential AmrZ targets and belonging to different functional classes, were tested for expression in a wild-type and an *amrZ*^*−*^ background. As shown in Figure 3B six of the tested genes including *flgZ*, *flhDC*, *fliC2*, PSF113_0118 and PSF113_4631 were clearly regulated by AmrZ under the experimental conditions tested (rich medium, exponential phase). Since *flhDC* encodes the master regulator for the production of the second flagellar apparatus of F113 [25], the results presented show that AmrZ regulates the production of both flagella. It is interesting to note that AmrZ regulates the expression of *flgZ*, which encodes a PilZ domain protein implicated in the regulation of motility and biofilm formation through sensing intracellular c-diGMP levels [34]. A negative result in this experiment does not necessarily means that these genes are not regulated by AmrZ, but that they do not show regulation under these conditions or that differences in expression are not statistically significant. It must be taken into account that identification of potential AmrZ targets by ChIP-seq was performed in cells that were ectopically overexpressing AmrZ. We have not found a relation between the score of the peaks or presence of the motif (Additional file 1) and regulation.

### AmrZ represses the expression of genes implicated in iron homeostasis in *P. fluorescens*

**Figure 4:**
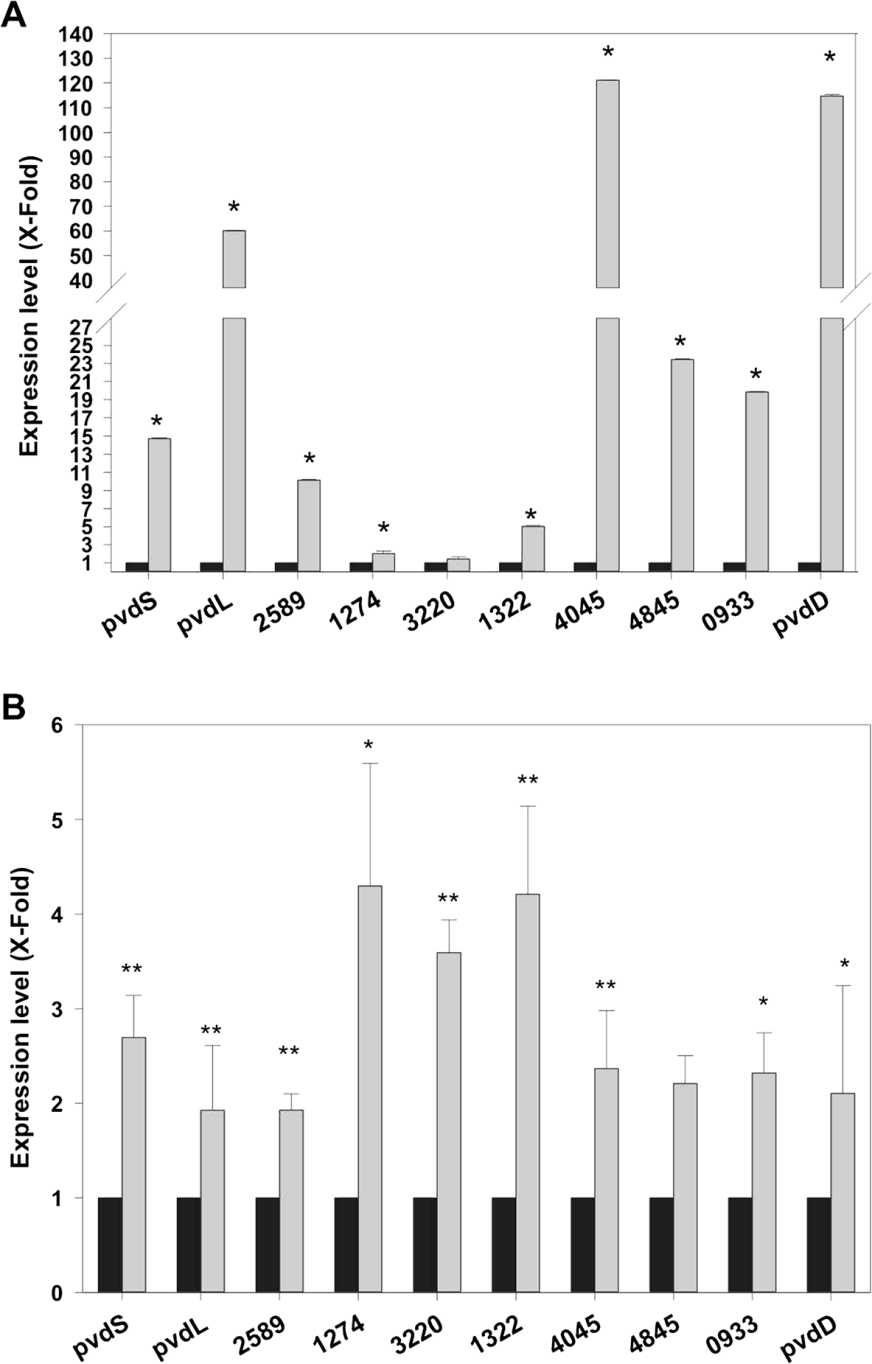
**AmrZ is a transcriptional repressor of genes implicated in iron homeostasis.** 10 genes potentially implicated in iron homeostasis and that appeared as enriched in the ChIP-seq analysis were selected for expression analysis (qPCR). **A**. Relative expression of the genes in *P. fluorescens* F113 after growth in iron sufficient medium (SA supplemented with 100 μM FeCl_3_, black bars) or iron limited medium (SA, gray bars). RNA was extracted from cells grown to 1.2 O.D._600_. Asterisks denotes statistically significant differences, p < 0.05. All genes appear to be induced under iron deficiency. **B**. Relative expression of the same genes in *P. fluorescens* F113 (black bars) and an *amrZ* mutant (gray bars). Numbers correspond to PSF113 annotation. RNA was extracted from cells grown under iron limiting conditions (SA medium) at 1.2 O.D._600_. Statistically significant differences are denoted by one (p < 0.1) or two asterisks (p < 0.05). All tested genes appear to be transcriptionally repressed by AmrZ.

Ten genes which were preceded by a peak and were predicted to encode genes involved in iron homeostasis were selected for further analysis. The selected genes include genes implicated in the production of the siderophore pyoverdine, ferrichrome and hemin receptors and extracytoplasmic sigma factors of the FecI family. In order to test iron dependence for transcription we analyzed the expression of these genes under iron limited conditions (SA medium) and iron sufficient conditions (SA medium supplemented with FeCl_3_). Since iron limitation is more evident at stationary phase (as judged by pyoverdine production by F113), RNA was collected at this stage. Figure 4A shows the relative expression of the ten genes. It can be observed that for all of the ten genes, expression was higher under iron limiting conditions. Differences were statistically significant (p < 0.05) for nine of the genes. These results show that most of the iron related genes putatively regulated by AmrZ are induced under iron limiting conditions. Expression of the ten iron related genes was also analyzed in an *amrZ*^*−*^ genetic background, under iron limiting conditions. As shown in Figure 4B, for all the ten genes, expression was higher in the *amrZ* mutant background. Differences were statistically significant for nine of the tested genes. Taken together, these results show that AmrZ is an important regulator of iron homeostasis in *P. fluorescens*. Under conditions where the stringent Fur repression has been removed [35], AmrZ may act at the transcriptional level as a negative regulator of iron homeostasis genes, including genes implicated in siderophore production and in collection of iron-siderophore complexes. The biological importance of AmrZ may be related to the metabolic saving by not overproducing siderophores, which are recyclable [36] and avoiding the toxicity imposed by hydroxyl anions because of an excessive iron import [37]. Regulation of iron homeostasis genes has been shown to be complex and dependent on multiple regulatory circuits. Besides the well-known repression through Fur during growth on iron sufficient conditions, it has been shown that in pseudomonads these genes are also regulated by the Gac/Rsm system and by c-diGMP levels [38]. The finding that AmrZ also regulates iron uptake related genes under specific conditions indicates the importance of this process for bacterial fitness and highlights the importance of fine tuning.

## Conclusions

The results presented here show that rather than a local regulator of motility and alginate production, AmrZ is a global regulator which may affect the transcription of hundreds of genes all along the chromosome. In the direct regulation of these genes, AmrZ acts mostly as a transcriptional repressor, as expected from its RHH domain architecture. AmrZ may also regulate gene expression indirectly through the regulation of genes encoding other transcriptional regulators and signal transduction proteins, notably proteins implicated in the turn-over of the messenger molecule c-diGMP. From the ontogeny of the AmrZ regulated genes and its own dependence on the sigma factor AlgU, it can be deduced the implication of AmrZ in regulating traits important for bacterial fitness and adaption to changing environments. The high degree of conservation across pseudomonads with very different lifestyles indicates the importance of AmrZ for environmental adaption.

Among the environment related traits directly regulated by AmrZ, is iron homeostasis. The results presented here show that a large number of genes implicated in iron uptake are repressed by AmrZ during growth under iron limiting conditions. These results show that AmrZ is a newly described regulator of iron homeostasis, which regulates iron uptake in conjunction with the Fur system, the Gac/Rsm pathway and the intracellular levels of c-diGMP.

## Methods

### Bacterial strains, plasmids, oligonucleotides and growth conditions

All *Pseudomonas fluorescens* strains used in this study are originated from the biocontrol strain F113 [39]. Oligonucleotides used in this study are listed in Additional file 2. The hemagglutinin peptide YPYDVPDYA (HA) was fused in-frame to AmrZ protein at both the N- terminal or the C-terminal by PCR using primers HAamrZF-amrZextR and amrZextF-amrZHAR, respectively. Both PCR fragments were cloned into the pGEM®-T Easy vector according to manufacturer’s instructions (Promega) and sequenced, and then subsequently cloned into the IPTG-inducible expression vector pVLT33 [40]. Plasmids were mobilized into *P. fluorescens* F113 by triparental mating using pRK600 as the helper plasmid [41]. The functionality of both AmrZ constructs was verified by complementation of the swimming motility phenotype of an *amrZ* mutant [22]. *P. fluorescens* strains were grown in SA medium (described as low-Fe medium in [42]) or LB medium [43] at 28°C; solid growth medium contained 1.5% (w/v) purified agar. When necessary, SA medium was supplemented with 100 μM FeCl_3_. *Escherichia coli* strains were grown in LB medium at 37°C. The following antibiotics were used, when required, at the indicated concentrations: rifampicin (Rif), 100 μg/mL; ampicillin (Amp), 100 μg/mL; kanamycin (Km), 25 μg/mL for *E. coli* or 50 μg/mL for *P. fluorescens*.

### Chromatin immunoprecipitation (ChIP) assay

To carry out this assay, 20 mL of culture from a *P. fluorescens* F113 strain harbouring the plasmid expressing either the N-terminal fusion protein HA-AmrZ or the C-terminal fusion protein AmrZ-HA was induced for 3 h with 1 mM IPTG. Cells were fixed with 1% formaldehyde for 10 min at room temperature and cross-linking was quenched by adding glycine to a final concentration of 120 mM. After washing the cells with ice-cold Phosphated-buffered saline (PBS), they were lysed in a non-ionic sonication buffer (50 mM Tris–HCl pH 8, 150 mM NaCl, 5 mM EDTA, 1% Triton X-100, 0.5% NP-40) containing protease inhibitor cocktail (Roche) and sonicated for 10 min in a Bioruptor™ UCD-200 TM (conditions: power H, 30 sec ON-30 sec OFF). Debris was removed by centrifugation, and the lysate was divided and immunoprecipitated with 6 μg of either anti-HA antibody (12CA5, Roche) or appropriate control IgG (sc-2025, Santa Cruz Biotechnology) and 30 μL of Dynabeads® protein G (Invitrogen). Immunocomplexes were washed three times with washing buffer (20 mM Tris–HCl pH 8, 2 mM EDTA, 1% Triton X-100, 0.1% SDS) containing an increasing concentration of NaCl (150–500 mM) and eluted from beads with elution buffer (25 mM Tris–HCl pH 8, 10 mM EDTA, 1% SDS). Immunoprecipitated DNA was released by reverting cross-linking at 65°C for 6 h and purified by Phenol:Chloroform:Isoamyl alcohol (25:24:1). The immunoprecipitated DNA was analyzed by qPCR and sequencing. Next generation sequencing was carried out by Illumina Genome Analyzer IIx single read, 75 bp each. Sequencing was performed at Servicio de Genómica, Parque Científico de Madrid. Raw sequence data has been deposited in NCBI SRA database (Bioproject accession number SRP039494).

### Gene expression analysis

Total RNA was extracted using Trizol® according to manufacturer’s specifications (Invitrogen) from the *P. fluorescens* F113 wild-type strain and its isogenic *amrZ* mutant [22]. Genomic DNA remains were removed by RQ1 RNase-Free DNase treatment (Promega) for 30 minutes at 37°C. After that, RNA was purified using Trizol®. The concentration of RNA was spectrophotometrically determined in a Nanodrop® and integrity was verified in denaturing agarose gels. All RNA samples were stored at -80°C.

RT-qPCRs were performed in two steps: a first step of cDNA synthesis using the iScript™ cDNA Synthesis Kit from Bio-Rad and a second step of qPCR using the FastStart Universal SYBR Green Master (Rox) from Roche. Gene expression was normalized by using 16S RNA as internal control. Every assay was performed three times with three replicates each time.

### Bioinformatic analysis

Illumina reads were clipped and trimmed to remove low quality nucleotides as well as putative Illumina adapters trails by using Trimmomatic [44], specifying a sliding window of 4 nts with an average phred quality of 20 and 50 nts as minimum read length to be conserved. Filtered reads were aligned to reference genome *P. fluorescens* F113 [GenBank: NC_016830] with Bowtie v2 [45]. Alignment sam file was analyzed by MACS v1.4 to detect reads enriched regions in the genome [46]. Reads histogram distribution and peak positions were introduced into a SQL database together with *P. fluorescens* F113 gene annotation. Enriched regions with a score, −10 · Log_10_ (p-value), equal or bigger than 1000 and a fold enrichment equal or bigger than 5.5 were selected for further study. A perl script was designed to extract nucleotide sequences 800 bps long, centered on selected peaks from reference genome and submitted to MEME [47] to detect conserved motifs with a maximum length of 20 nts.

InterPro and Gene Ontology semantic values were used to assign functional categories to genes close to enriched regions.

### Statistical analysis

SigmaStat software package (Systat software) was used for all statistical analyses. The data were compared using Student’s t-test for independent samples (p < 0.05 or p < 0.1).

### Availability of supporting data

The raw sequence data set supporting the results of this article is available in the NCBI SRA repository, Bioproject accession number SRP039494, http://www.ncbi.nlm.nih.gov/sra/?term=SRP039494.

The following additional data are available with the online version of this paper. Additional data file 1 is a table listing the genes that correspond to the peaks from the ChIP-seq. For each peak, the score, overrepresentation, presence of the conserved motif and the p value of the motif, is given. Additional data file 2 is a table listing the oligonucleotides used in this study.

## Abbreviations

RHH: Ribbon-Helix-Helix; ChIP: Chromatin immunoprecipitation; qPCR: quantitative or real time PCR; c-diGMP: cyclic diguanylate monophosphate; MEME: Multiple Em for Motif Elicitation; HA: Hemagglutinin peptide; MACS: Model-based Analysis of ChIP-Seq

## Competing interests

The authors declare that they have no competing interests.

## Authors’ contributions

FM-G participated in study design, performed immunoprecipitation and expression experiments, participated in writing the Ms. MR-N carried out bioformatic analysis and participated in writing the Ms. PV performed gene expression analysis. MM and RR conceived and supervised the study, and participated in writing the Ms. All authors read and approved the final Ms.

## Supplementary Material

Additional file 1 **ChIP-Seq peaks.** Nearest gene, score, overrepresentation, presence of the conserved motif and its p value are given for every peak. Click here for file

Additional file 2 **Oligonucleotides used.** List and sequence of primers used for ChIP, q-PCR and AmrZ fusions. Click here for file
