# Supplementary material for: AmrZ is a global transcriptional regulator implicated in iron uptake and environmental adaption in P. fluorescens F113
Source: BMC Genomics. 2014 Mar 26;15:237. doi: 10.1186/1471-2164-15-237 (PMC3986905; doi:10.1186/1471-2164-15-237)
Supplement: Additional file 1 — ChIP-Seq peaks. Nearest gene, score, overrepresentation, presence of the conserved motif and its p value are given for every peak. [file 1471-2164-15-237-S1.pdf]

Locus	Gene	Product	Score	Fold Enrichment	Motif	Motif p-value
**Motility/Chemotaxis**						
PSF113_0118	*cheY8*	CheY	1442,62	8,61	Yes	6,16E-09
PSF113_0374	-	Methyl-accepting chemotaxis protein	3100	9,53	Yes	9,41E-07
PSF113_0569	-	Methyl-accepting chemotaxis protein	1503,09	6,94	Yes	1,27E-05
PSF113_0660	-	Methyl-accepting chemotaxis protein	1501,28	6,73	Yes	6,36E-07
PSF113_0740	*fliC2*	FliC2	1414,26	6,03	No	N.A.
PSF113_0751	*flhD*	FlhD	3100	8,28	Yes	7,26E-07
PSF113_0781	*flgM2*	FlgM2	3100	9,06	Yes	1,34E-07
PSF113_0882	-	Methyl-accepting chemotaxis protein	3047,07	9,51	Yes	1,04E-05
PSF113_1675	-	Methyl-accepting chemotaxis protein	1797,39	7,19	Yes	7,22E-08
PSF113_1738	*cheV*	CheV	3100	11,84	Yes	2,10E-07
PSF113_2159	-	Methyl-accepting chemotaxis protein	3100	9,7	Yes	6,36E-07
PSF113_2441	-	Methyl-accepting chemotaxis protein	3100	10,52	Yes	9,91E-08
PSF113_2521	-	Methyl-accepting chemotaxis protein	3111,39	8,68	Yes	3,19E-08
PSF113_2586	-	Methyl-accepting chemotaxis protein	3100	7,61	Yes	3,63E-05
PSF113_2925	-	Methyl-accepting chemotaxis protein	3100	9,78	Yes	3,16E-06
PSF113_3014	*cheA4*	CheA	1085,4	6,48	No	N.A.
PSF113_3554	-	Methyl-accepting chemotaxis protein	3100	6,57	Yes	4,95E-06
PSF113_3817	*cheY6*	CheY	1246,93	6,13	No	N.A.
PSF113_4205	-	Methyl-accepting chemotaxis protein	3130,58	8,04	Yes	3,23E-07
PSF113_4460	*flgZ*	FlgZ	1771,83	7,03	Yes	1,89E-05
PSF113_4941	-	Methyl-accepting chemotaxis protein	3100	6,84	Yes	1,98E-06
**Iron**						
PSF113_0933	*fagA*	FagA	3133,59	7,47	Yes	3,63E-05
PSF113_1274	-	TonB-dependent hemin , ferrichrome receptor	3100	12,45	Yes	3,03E-05
PSF113_1322	-	Iron-regulated protein A precursor	3100	10,6	Yes	1,72E-05
PSF113_1749	*pvdS*	PvdS	3100	10,64	No	N.A.
PSF113_1750	*pvdL*	PvdL	3100	10,64	No	N.A.
PSF113_1837	*pvdD*	PvdD	1051,57	5,99	No	N.A.
PSF113_1856	-	Outer membrane pyoverdine eflux protein	3100	5,99	No	N.A.
PSF113_2258	-	Outer membrane ferripyoverdine receptor	2248,65	8,34	Yes	1,72E-05
PSF113_2454	-	RNA polymerase sigma-70 factor, ECF subfamily	2270,92	8,09	No	N.A.
PSF113_2589	-	Ferrichrome-iron receptor	3100	12,04	No	N.A.
PSF113_3151	-	Ferrichrome-iron receptor	3100	9,6	Yes	7,62E-06
PSF113_3220	-	Heme uptake regulator	3100	9,9	No	N.A.
PSF113_3734	-	Ferrichrome-iron receptor	3100	11,17	Yes	1,72E-05
PSF113_4045	-	Iron-regulated membrane protein	3100	9,58	Yes	7,26E-05
PSF113_4568	-	Bacterioferritin-associated ferredoxin	3100	10,09	Yes	6,13E-05
PSF113_4569	-	Bacterioferritin	3100	10,09	Yes	6,13E-05
PSF113_4845	-	RNA polymerase sigma-70 factor, ECF subfamily	2465,83	7,17	No	N.A.
PSF113_5412	*fiuA*	FiuA	3100	9,78	No	N.A.
PSF113_5657	*fbpA*	FbpA	1075,78	8,34	No	N.A.

**Regulation/Signal transduction**							
	PSF113_0361	*ntrB*	NtrB	2573,18	5,96	No	N.A.
	PSF113_0661	-	Putative two-component response regulator	2205,29	7,1	Yes	1,07E-06
	PSF113_1200	-	LysR family transcriptional regulator	3012,26	5,78	No	N.A.
	PSF113_1413	*algU*	AlgU	1721,41	6,4	Yes	4,25E-07
	PSF113_1768	*sigX*	SigX	2211,04	6,6	Yes	7,26E-05
	PSF113_2913	-	Transcriptional repressor, LacI family	2493,83	9,66	Yes	4,43E-06
	PSF113_3565	-	Sensory box histidine kinase	3100	10,02	Yes	9,91E-08
	PSF113_3566	-	Signal transduction histidine kinase	3100	10,02	Yes	9,91E-08
	PSF113_3957	*staS*	StaS	1107,42	6,2	Yes	6,16E-06
	PSF113_3958	*rsbU2*	RsbU	1107,42	6,2	Yes	6,16E-06
	PSF113_4024	-	Transcriptional regulator, Cro/CI family	1450,05	6,33	No	N.A.
	PSF113_4193	-	Two-component response regulator	1864,32	6,87	Yes	9,91E-08
	PSF113_4470	*amrZ*	AmrZ	3100	7,08	Yes	2,51E-05
	PSF113_4596	*cspA3*	CspA	3100	9,14	Yes	1,21E-06
	PSF113_4631	-	Sensory box histidine kinase	3100	8,65	Yes	3,63E-05
	PSF113_4680	-	Two-component response regulator	3100	5,96	Yes	2,51E-05
	PSF113_4807	-	Transcriptional regulator containing an amidase domain and an AraC-type DNA-binding HTH domain	1189,25	7,08	No	N.A.
	PSF113_4873	-	PAS PAC sensor protein	2106,22	6,66	No	N.A.
	PSF113_5078	-	Autoinducer-binding transcriptional family	1283,34	6,92	Yes	3,23E-07
	PSF113_5133	*ladS*	LadS	3100	7,01	Yes	1,56E-05
	PSF113_5263	-	ATP-dependent transcriptional regulator containing adenylate cyclase related domains	3100	10,18	Yes	2,50E-06
	PSF113_5334	*vfr*	Vfr	3052,54	7,67	Yes	3,97E-05
	PSF113_5349	-	Putative two-component sensor, near polyamine transporter	2329,89	6,48	Yes	5,17E-05
	PSF113_5523	-	Transcriptional regulator	3157,07	5,51	No	N.A.
	PSF113_5782	*sadB*	SadB	3100	6,5	Yes	2,23E-06
**c-di-GMP**							
	PSF113_0499	-	Sensory box-containing diguanylate cyclase	2105,9	5,56	Yes	3,54E-06
	PSF113_0714	*yfiR*	YfiR	3100	9,32	Yes	1,21E-06
	PSF113_1982	-	GGDEF domain protein	3100	8,43	Yes	4,25E-07
	PSF113_2333	-	Sensory box/GGDEF family protein	2773,34	8,08	Yes	2,44E-07
	PSF113_3553	-	Metal dependent phosphohydrolase	3100	6,57	Yes	4,95E-06
	PSF113_4023	-	Diguanylate cyclase phosphodiesterase with PAS PAC sensor	1450,05	6,33	No	N.A.
	PSF113_4038	-	PAS PAC sensor-containing diguanylate cyclase	3031,29	9,15	Yes	1,81E-07
	PSF113_4360	-	EAL domain/GGDEF domain protein	3100	11,74	Yes	2,44E-07
	PSF113_4681	-	Diguanylate cyclase phosphodiesterase with PAS PAC sensor	3100	5,96	Yes	2,51E-05
	PSF113_4776	-	GGDEF domain protein	3100	6,03	Yes	9,41E-07
	PSF113_4827	-	Diguanylate cyclase/phosphodiesterase (GGDEF & EAL domains) with PAS/PAC sensor(s)	2590,03	7,34	No	N.A.
	PSF113_5064	-	Sensory box GGDEF domain EAL domain protein	3100	9,69	Yes	3,71E-07
	PSF113_5392	-	GGDEF domain protein	2400,89	8,84	Yes	2,81E-07
**Virulence**							
	PSF113_1489	-	Hemolysin activator protein precursor	3100	12,27	Yes	8,47E-08
	PSF113_1855	-	RHS repeat-associated core domain-containing protein	3100	5,99	No	N.A.

	PSF113_2273	-	Reticulocyte binding protein	1598,26	6,63	Yes	4,43E-06
	PSF113_2409	*vgrG*	VgrG	1429,5	6,15	No	N.A.
	PSF113_2459	*phlD*	PhlD	1308,69	5,53	Yes	1,89E-05
	PSF113_2737	-	Hemolysin-type calcium binding protein	1766,48	7,11	Yes	6,36E-07
	PSF113_4636	*secA*	SecA	3100	6,09	Yes	2,29E-05
	PSF113_5796	*impI*	ImpI	3100	8,57	Yes	3,71E-07
	PSF113_5797	*impA2*	ImpA	3100	8,57	Yes	3,71E-07
**Cell Wall**							
	PSF113_0208	*lapA*	LapA	2119,27	6,57	Yes	3,71E-07
	PSF113_0393	*mdoG*	MdoG	3100	9,42	Yes	1,56E-05
	PSF113_1188	*rfaZ*	RfaZ	1005,83	6,13	Yes	3,96E-06
	PSF113_1189	-	Chitinase class I family protein	1005,83	6,13	Yes	3,96E-06
	PSF113_4752	*algD*	AlgD	3100	9,42	Yes	7,26E-05
	PSF113_5150	-	Muramoyltetrapeptide carboxypeptidase	3100	6,49	No	N.A.
**Transport**							
	PSF113_0209	-	Type I secretion outer membrane family	2119,27	6,57	Yes	3,71E-07
	PSF113_0236	-	Methionine ABC transporter substrate-binding protein	3100	9,32	Yes	6,67E-05
	PSF113_0344	-	Oxalate/formate antiporter	2868,7	7,1	Yes	2,50E-06
	PSF113_0683	*copP*	CopP	1029,8	5,69	No	N.A.
	PSF113_0894	*proW2*	ProW	1607,44	6,6	No	N.A.
	PSF113_3190	-	CAAX amino terminal protease family protein	2260,52	8	Yes	9,39E-06
	PSF113_4094	-	Major facilitator family transporter	2242,84	6,11	Yes	1,09E-04
	PSF113_5063	-	ABC transporter, ATP-binding protein	3100	9,69	Yes	3,71E-07
	PSF113_5474	-	ABC-type amino acid transport/signal transduction systems, periplasmic component/domain protein	3100	7,68	Yes	3,78E-08
**Others**							
	PSF113_0079c	-	Phage-related replication protein-like protein	1469,27	5,55	No	5,63E-05
	PSF113_0093	*cynT*	CynT	1796,72	5,83	Yes	2,29E-05
	PSF113_0225	-	Microcystin dependent protein	2426,84	7,91	Yes	2,50E-06
	PSF113_0343	-	Acetyltransferase	2868,7	7,1	Yes	1,56E-05
	PSF113_0392	*dtd*	Dtd	3100	9,42	Yes	2,76E-05
	PSF113_0429	*aroB*	AroB	1984,44	6,11	Yes	4,87E-07
	PSF113_0730	-	Glyoxalase family protein	3100	9,21	Yes	N.A.
	PSF113_0889	-	Flavodoxin nitric oxide synthase	3100	10,56	No	N.A.
	PSF113_0895	*prfC*	PrfC	1607,44	6,6	No	N.A.
	PSF113_0929	-	Nitrilase cyanide hydratase and apolipoprotein N-acyltransferase	1214,9	6,56	No	N.A.
	PSF113_0930	*tldD*	TldD	1214,9	6,56	No	2,08E-05
	PSF113_0964	-	Medium-chain-fatty-acid-CoA ligase	1884,91	7,84	Yes	9,39E-06
	PSF113_0976	-	Phage protein	2998,54	8,76	Yes	N.A.
	PSF113_1047	-	Multicopper oxidase	1633,68	7,08	No	1,89E-05
	PSF113_1150	-	tRNA(Ile)-lysidine synthetase	2193,87	7,01	Yes	1,89E-05
	PSF113_1151	*pyrG*	PyrG	2193,87	7,01	Yes	2,08E-05
	PSF113_1177	-	Prophage long tail fiber protein H	2784,36	5,5	Yes	2,08E-05
	PSF113_1178	-	Prophage tail fimber assembly protein	2784,36	5,5	Yes	N.A.

	PSF113_1201	-	Ferredoxin--NADP(+) reductase	3012,26	5,78	No	1,18E-04
	PSF113_1295	-	NAD-dependent aldehyde dehydrogenase	1509,92	5,77	Yes	4,25E-07
	PSF113_1412	*nadB*	NadB	1721,41	6,4	Yes	4,95E-06
	PSF113_1877	-	Propeptide amd peptidase m4	1873,51	6,97	Yes	N.A.
	PSF113_2031	*pcpA*	PcpA	3210,32	6,77	No	N.A.
	PSF113_2126	-	Dihydrodipicolinate synthase	1344,1	5,84	No	6,36E-07
	PSF113_2158	*nuoA*	NuoA	3100	9,7	Yes	2,44E-07
	PSF113_2332	-	Pyrazinamidase nicotinamidase	2773,34	8,08	Yes	N.A.
	PSF113_2569	-	Carbon starvation protein A	1853,85	6,08	No	3,16E-06
	PSF113_2926	-	Aminotransferase class I and II	3100	9,78	Yes	4,34E-05
	PSF113_2972	-	Putative membrane protein	2374,87	9,1	Yes	4,43E-06
	PSF113_3038	-	NTP pyrophosphohydrolaseincluding oxidative damage repair enzyme	3100	9,51	Yes	N.A.
	PSF113_3409	-	Acyl-CoA dehydrogenase, long-chain specific	1302,86	7,26	No	1,15E-05
	PSF113_3711	-	Valyl-tRNA synthetase	3100	7,65	Yes	6,85E-06
	PSF113_3789	-	Ferredoxin	1914,84	6,92	Yes	1,75E-06
	PSF113_3853	-	Contains type I hydrophobic transmembrane region and ATP/GTP binding motif	2183,08	7,01	Yes	1,41E-05
	PSF113_3877	-	Carboxyphosphonoenolpyruvate phosphonomutase	2040,09	9,89	Yes	6,85E-06
	PSF113_3889	-	Zinc carboxypeptidase domain protein	3100	9,76	Yes	3,23E-07
	PSF113_3891	-	Catalase	1622,51	5,57	Yes	N.A.
	PSF113_3918	*tig*	Tig	2702,4	6,91	No	N.A.
	PSF113_3922	*folD*	FolD	2702,4	6,91	No	2,44E-07
	PSF113_3975	*mfd*	Mfd	1445,57	5,86	Yes	N.A.
	PSF113_4083	-	Sterol desaturase	1771,44	7,37	No	1,09E-04
	PSF113_4096	-	Enoyl-CoA hydratase/isomerase family protein	2242,84	6,11	Yes	3,97E-05
	PSF113_4101	-	3-ketoacyl-CoA thiolase, Acetyl-CoA acetyltransferase	2040,81	7,09	Yes	3,97E-05
	PSF113_4102	-	Cytochrome c, mono- and diheme variant	2040,81	7,09	Yes	1,27E-04
	PSF113_4137	*moaA2*	MoaA	2910,49	6,18	Yes	3,63E-05
	PSF113_4154	-	Haloacid dehalogenase-like hydrolase	1283,28	6,23	Yes	3,63E-05
	PSF113_4155	-	Ribosomal large subunit pseudouridine synthase C	1283,28	6,23	Yes	9,91E-08
	PSF113_4192	-	Flp fap pilin component	1864,32	6,87	Yes	N.A.
	PSF113_4272	-	Chorismate synthase	1322,14	6,71	No	N.A.
	PSF113_4273	-	Hydrolase, alpha/beta fold family	1322,14	6,71	No	2,23E-06
	PSF113_4280	-	DNA mismatch repair protein	3100	7,51	Yes	4,43E-06
	PSF113_4410	-	Cobyrinic acid-diamide adenosyltransferase	3100	8,62	Yes	1,37E-06
	PSF113_4529	-	PHP-like protein	3100	7,51	Yes	1,21E-06
	PSF113_4595	-	Aminomethyltransferase (glycine cleavage system T protein)	3100	9,14	Yes	N.A.
	PSF113_4731	-	Ketosteroid isomerase-related protein	3100	8,34	No	N.A.
	PSF113_4806	-	Butyryl-CoA dehydrogenase	1189,25	7,08	No	N.A.
	PSF113_4872	*yqcA*	YqcA	2106,22	6,66	No	N.A.
	PSF113_4932	*prs*	Prs	3181,2	5,84	No	1,98E-06
	PSF113_4940	-	Fosmidomycin resistance protein	3100	6,84	Yes	3,54E-06
	PSF113_4950	-	Putative exported protein	3100	9,72	Yes	5,53E-06
	PSF113_4978	-	Pentapeptide repeat-containing protein	3100	7,85	Yes	N.A.

	PSF113_5071	-	Carbon starvation protein A	3100	5,73	No	1,56E-05
	PSF113_5134	*thiD*	ThiD	3100	7,01	Yes	3,97E-05
	PSF113_5333	-	OsmC/Ohr family protein	3052,54	7,67	Yes	3,78E-08
	PSF113_5475	-	Acetyltransferase	3100	7,68	Yes	N.A.
	PSF113_5686	*hemB*	HemB	1573,37	6,67	No	N.A.
	PSF113_5687	-	DedA family protein	1573,37	6,67	No	N.A.
**Unknown**							
	PSF113_0226	-	Hypothetical protein	2426,84	7,91	Yes	2,29E-05
	PSF113_0370	-	Putative membrane protein	1833,27	7,18	No	N.A.
	PSF113_0375	-	Putative lipoprotein	3100	9,53	Yes	9,41E-07
	PSF113_0580	-	Hypothetical protein	3100	9,97	Yes	3,23E-07
	PSF113_0662	-	Hypothetical protein	2205,29	7,1	Yes	1,07E-06
	PSF113_0682	-	Hypothetical protein	1029,8	5,69	No	N.A.
	PSF113_0876	-	Hypothetical protein	3100	9,07	Yes	1,37E-06
	PSF113_0877	-	Hypothetical protein	3100	9,07	Yes	1,37E-06
	PSF113_0916	-	Hypothetical protein	3100	11,58	Yes	6,36E-07
	PSF113_0975	-	Hypothetical protein	2998,54	8,76	Yes	9,39E-06
	PSF113_1104	-	Hypothetical protein	3100	11,33	Yes	1,34E-07
	PSF113_1294	-	Hypothetical protein	1509,92	5,77	Yes	1,18E-04
	PSF113_1304	-	Putative perforin-like protein 2	1458,9	7,25	Yes	5,17E-05
	PSF113_1488	-	Hypothetical protein	3100	12,27	Yes	8,47E-08
	PSF113_1729	-	Putative lipoprotein	2172,68	5,84	Yes	5,63E-05
	PSF113_1737	-	Hypothetical protein	3100	11,84	Yes	2,10E-07
	PSF113_1876	-	Hypothetical protein	1873,51	6,97	Yes	4,95E-06
	PSF113_1941	-	Putative membrane protein	2177,97	7,77	Yes	6,18E-08
	PSF113_1942	-	Frg domain protein	2177,97	7,77	Yes	6,18E-08
	PSF113_1981	-	Hypothetical protein	3100	8,43	Yes	4,25E-07
	PSF113_2129	-	Hypothetical protein	3100	10,79	Yes	4,87E-07
	PSF113_2272	-	Hypothetical protein	1598,26	6,63	Yes	4,43E-06
	PSF113_2448	-	Hypothetical protein	3100	10,61	Yes	1,89E-05
	PSF113_2522	-	Hypothetical protein	3111,39	8,68	Yes	3,19E-08
	PSF113_2587	-	Putative membrane protein	3100	7,61	Yes	3,63E-05
	PSF113_2952	-	Hypothetical protein	3100	9,36	Yes	1,41E-05
	PSF113_3013	-	Putative cell wall-associated hydrolase	1085,4	6,48	No	N.A.
	PSF113_3039	-	Hypothetical protein	3100	9,51	Yes	4,43E-06
	PSF113_3171	-	Hypothetical protein	1806,81	5,98	Yes	4,95E-06
	PSF113_3258	-	Hypothetical protein	3100	8,34	Yes	3,03E-05
	PSF113_3296	-	Hypothetical protein	2741,95	6,51	Yes	2,29E-05
	PSF113_3652	-	Putative exported protein	3100	9,49	Yes	1,89E-05
	PSF113_4106	-	Putative superfamily II DNA/RNA helicase, SNF2 family	1495,88	6,54	Yes	6,16E-06
	PSF113_4204	-	Putative membrane protein	1725,3	6,64	Yes	3,71E-07
	PSF113_4318	-	Hypothetical protein	2229,39	8,06	Yes	3,03E-05
	PSF113_4319	-	Hypothetical protein	2229,39	8,06	Yes	3,03E-05

	PSF113_4411	-	Hypothetical protein	3100	8,62	Yes	4,43E-06
	PSF113_4637	-	Hypothetical protein	3100	6,09	Yes	2,29E-05
	PSF113_5053	-	Hypothetical protein	1244,93	7,43	Yes	1,41E-05
	PSF113_5194	-	Putative membrane protein	3100	8,26	Yes	9,41E-07
	PSF113_5264	-	Hypothetical protein	3100	10,18	Yes	2,50E-06
	PSF113_5350	-	Hypothetical protein	2329,89	6,48	Yes	5,17E-05
	PSF113_5423	-	Hypothetical protein	2575,11	7,8	Yes	9,28E-05
	PSF113_5424	-	Hypothetical protein	2575,11	7,8	Yes	9,28E-05
	PSF113_5504	-	Putative membrane protein	1812,58	6,89	Yes	2,51E-05
	PSF113_5783	-	Putative superfamily II DNA and RNA helicase	3100	6,5	Yes	2,23E-06
