# Supplementary material for: AmrZ is a global transcriptional regulator implicated in iron uptake and environmental adaption in P. fluorescens F113
Source: BMC Genomics. 2014 Mar 26;15:237. doi: 10.1186/1471-2164-15-237 (PMC3986905; doi:10.1186/1471-2164-15-237)
Supplement: Additional file 2 — Oligonucleotides used. List and sequence of primers used for ChIP, q-PCR and AmrZ fusions. [file 1471-2164-15-237-S2.pdf]

**Locus ID**	**ChIP (5´-3´)**	**RT-qPCR (5´-3´)**
PSF113_1559 *(fleQ*)	Fw: AGGCGTCTACAGGCATGACAA	
	Rv: TTTAAGATCACCGCCAAGTCG	
PSF113_4752 *(algD*)	Fw: TGCACTATGACCGCTGAACAC	
	Rv: GCGACATCTACGCCAACTACG	
PSF113_4470 (*amrZ*)	Fw: CATTTCCGTTATCCGCCAGAC	
	Rv: TCATGCCTCAAAATTGCCAGA	
PSF113_0714 (*yfiR*)	Fw: GGGTTGATGCTGGAGTTGGAG	
	Rv: CCAGCGAGACAGCGTGTTATG	
PSF113_4360	Fw: ATGGTGATGCTCGTCGGAGT	Fw: GCGCATCGTGGGTTATGAAG
	Rv: CTGTCGTGGTCATGGCAATCT	Rv: CCAGTTCCAATTCGCTGAGG
PSF113_5392	Fw: CGATGGCGTTTGTTCAGTCAG	Fw: CGACGCTGGTGTTGTTGCT
	Rv: GAGGGGACGGTTTTCTTAGGG	Rv: CCAGGAGGAAGTAGGCGATGT
PSF113_1274	Fw: GATAGGCTCCGCTGATTCGTC	Fw: CCACCTGAAAAAGCCCTGGA
	Rv: GCAAGAAACCGGCAAGTAAGG	Rv: GCGCTGATGGTCACTTGTTG
PSF113_1322	Fw: TACCGAAGGCGGCAATCTC	Fw: GGCGAAGGCAAAGACAAGG
	Rv: ATCATGGCGAACATCCTGCTT	Rv: TAGACGATGTCGGCGTAGTGC
PSF113_1749 (*pvdS*)	Fw: TCGCACCTGCTTGTGGATACT	Fw: CGCCAGAAACCTCCCATATCA
	Rv: CTAATTTGGCGCTCGATGTGT	Rv: GCGGTACATCTCGAAGGCATAG
PSF113_1750 (*pvdL*)	Fw: TCGCACCTGCTTGTGGATACT	Fw: AGGCAGGAAACCCCCATGAC
	Rv: CTAATTTGGCGCTCGATGTGT	Rv: CGCGATAACTCAACACCACAC
PSF113_4569	Fw: TTCAAGGAACCGGACTCAATGT	Fw: GAGATGCTTGAGTGCGACCTG
	Rv: GCGCATTTACGAGTGTCAAACC	Rv: CGATGTGCTCTTCCTCGGATT
PSF113_0750-0751 (*flhDC*)	Fw: GGAACTATCCGCCAAGTACGA	Fw: TGGAACAGGTCGGTCATCAAG
	Rv: TCACATACTCGACCCTGCTCAA	Rv: GCGTGAGAGTGAGCAACGAA
PSF113_3554	Fw: ATAGTTTCGCGGCCATCACTT	Fw: GTGTGGAGCCTGCGTTTATTG
	Rv: TTGCCTGTCCTTTCACCTTCA	Rv: CCTCACCCAATCCTTCATCAG
PSF113_4205	Fw: ACTGGCTACGGATCAATGTGG	Fw: GGATTTGTCCTGGTGCTGCT
	Rv: AAGATGCTGGCGTCGGAATAA	Rv: GAGGGCCGACATCTGTTTCA
PSF113_4460 (*flgZ*)	Fw: GCCAGTCGATCCTGATGCAA	Fw: CCGCGAGACCAACTCCATT
	Rv: ATCATCCGCGTTTGAGGCA	Rv: GTCAGTGGACCCTTGCCATC
PSF113_0118	Fw: TGCCATCAGTAAAAGCGGTATG	Fw: TGCTTCGTTCATTCGTGACCT
	Rv: CCAGGTCACGAATGAACGAAG	Rv: GGCATTTCCCAATCGCACA
PSF113_5133 (*ladS*)	Fw: TGACACTTATTGGGGCTGCTTC	Fw: CTCTCGATCAGCGTTCTGGTT
	Rv: GTTGCCTTGGTCAGGTCGTAA	Rv: CGCATCGGGTAGGTACAAGTC
PSF113_5263	Fw: ACAGTCCAGAGCGGTGTCAGT	Fw: ATGGACTTGCGACCAAACACC
	Rv: GTTTGGTCGCAAGTCCATGTC	Rv: GCAGCCCTTTCAACGACAC
PSF113_5333	Fw: CTTCATCGCCAAGACTCCACT	Fw: AAGCCATGTTCCTCGGTGAGT
	Rv: ATGCTGGTCGGTCAGGTCTC	Rv: CCCAGCAAGAGCATTTCCATC
PSF113_5334 (*vfr*)	Fw: CTTCATCGCCAAGACTCCACT	Fw: GCCATCCAGCCAAACACAAC
	Rv: ATGCTGGTCGGTCAGGTCTC	Rv: ACCGTCGTCATCTTCTATCAGG
PSF113_2409 (*vgrG*)	Fw: CCAGAATGGAAACGCCAATAA	Fw: GACAGCAACACCACGCTATCA
	Rv: CGGTGAGTGATAGCGTGGTGT	Rv: CGTTGAGGATGTGCGAACC
dif region	Fw: TTGAAGCCAATCCCATCCTCT	
	Rv: TTCGTTCCTTCCTTGTTGTCG	
PSF113_4631		Fw: TGTTGTTGACCACTGTCCTGCT
		Rv: CGGAGATCAGGCTTGGATTG
PSF113_5797 (*impA*)		Fw: GTGGATGTGCCTTTGTTGCTC
		Rv: CATGCTACGTTCGGGCTGAC
PSF113_1837 (*pvdD*)		Fw: GACAAGGAGGGCGTGGAGTT

Locus ID	ChIP (5´-3´)	RT-qPCR (5´-3´)
		Rv: CAGGTTGTAAGCACCGCTGTC
PSF113_0889		Fw: CGACCGTGTTCTACCTGCTGA
		Rv: ACTGCGCCTTGAAGCTCTTGT
PSF113_2589		Fw: TGTTCAGAAATGGGCATCCAG
		Rv: TCCTCGCTCAGCTCATACTCG
PSF113_3220		Fw: TCCGCCAACGATCTGTCTCTA
		Rv: ACGTATCCTGGGCAAGGTCTG
PSF113_4045		Fw: CTTCGGTTTGGTCCTGAGCAT
		Rv: GGGTTTCTGGCTACGCTTGAG
PSF113_4845		Fw: GAAGAGGAACGGGCAATCATC
		Rv: TGACCGTCGCCAATGAAATAC
PSF113_0933		Fw: CACTGCGCGACCTCGAACAAC
		Rv: TACTCATCGGGAGATCCTTGC
PSF113_1554 (*fliC*)		Fw: TTCCGATGCTCTGTCCACTTC
		Rv: GATACCGTCGTTGGCGTTCTT
PSF113_0740 (*fliC2*)		Fw: GACCAGACCATCACCATCCAG
		Rv: AGCGTTGTGTCATAGCCGTTC

**AmrZ fusions (5´-3´)[a]**	
HAamrZF	ATGTCTTATCCATACGATGTTCCAGATTATGCTCGCCCATTGAAACAGGCA
amrZextR	TCAGGTCGCGTCTGCGG
amrZextF	ATGCGCCCATTGAAACAGG
amrZHAR	TTAAGCATAATCTGGAACATCGTATGGATAGGTCGCGTCTGCGGCCA

[a]HA sequence is underlined
